# Serum pro-B-type natriuretic peptide levels and cardiac index as adjunctive tools of blunt cardiac injury

**DOI:** 10.1186/s12872-022-02990-2

**Published:** 2023-02-10

**Authors:** Chae-Min Bae, Joon Yong Cho, Hanna Jung, Shin-Ah Son

**Affiliations:** Department of Thoracic and Cardiovascular Surgery, School of Medicine, Kyungpook National University, Kyungpook National University Hospital, 130 Dongdeok-ro, Jung-gu, Daegu, Republic of Korea

**Keywords:** Multiple trauma, Heart injuries, Intensive care unit, Fractures

## Abstract

**Background:**

Blunt cardiac injury (BCI) has a variety of symptoms that may be a potentially life-threatening injury that can lead to death. Depending on the diagnosis of BCI, treatment direction and length of stay may vary. In addition, the utility of other diagnostic tests for cardiac disease as diagnostic tools for BCI remain unclear. The purpose of this study was to investigate the competence of N-terminal pro-B-type natriuretic peptide (NT pro-BNP) and cardiac index (C.I) as adjunctive diagnostic tools for BCI.

**Methods:**

From January 2018 to March 2020, severe trauma patients with sternum fracture who were admitted to the traumatic intensive care unit (TICU) were included this study. Patients with sternum fracture, 18 years of age or older, and with an injury severity score > 16 who required intensive care were included. Invasive measurement for the analysis of the pulse contour for C.I monitoring and intravenous blood sampling for NT pro-BNP measurement were performed. Sampling and 12-lead electrocardiogram were performed at different time points as follows: immediately after TICU admission and at 24 h and 48 h after trauma.

**Results:**

Among 103; 33 patients with factors that could affect NT pro-BNP were excluded; therefore, 63 patients were included in this study. According to the American Association for the Surgery of Trauma Cardiac Injury Scale, 33 patients were diagnosed with non-BCI, and 30 patients constituted with BCI. The median ages of the patients were 58 (52–69), and 60 (45–69) years in the non-BCI and BCI groups, respectively (*p* = 0.77). The median NT pro-BNP values were higher in the BCI group on admission, hospital day (HD) 2, and HD 3, however, no statistical difference was observed (125 (49–245) vs. 130 (47–428) pg/mL, *p* = 0.08, 124 (68–224) vs*.* 187 (55–519) pg/mL*, p* = 0.09, and 121(59–225) vs. 133 (56–600) pg/mL, *p* = 0.17, respectively). On the contrary, significantly lower values were observed in the median C.I measurement on admission and HD 3 in the BCI group (3.2 (2.8–3.5) vs*.* 2.6 (2.3–3.5) L/min/m^2^, *p* < 0.01 and 3.2 (3.1–3.9) vs*.* 2.9 (2.4–3.2) L/min/m^2^, *p* < 0.01, respectively); however, no significant difference was observed on HD 2 (3.4 (3.0–3.7) vs*.* 2.6 (2.4–3.4) L/min/m^2^, *p* = 0.17), Furthermore, The median lactate levels in the BCI group upon admission, HD 2, and HD 3 were significantly higher than those in the non-BCI group (1.8 (1.1–2.6) vs*.* 3.1 (2.1–4.4) mmol/L*, p* < 0.01; 1.3 (0.8–2.3) vs*.* 3.0 (2.2–4.7) mmol/L, *p* < 0.01; and 1.5 (0.9–1.5) vs*.* 2.2 (1.3–3.7) mmol/L, *p* < 0.01, respectively).

**Conclusion:**

Consecutive values of NT pro-BNP and C.I show no correlation with ECG-based BCI diagnosis. However, lactate level measurement may help in the early recognition of BCI as an adjunctive tool. It should be noted that this is a hypothesis-generating study for BCI diagnosis. Further studies should be conducted in larger populations with a prospective approach.

**Supplementary Information:**

The online version contains supplementary material available at 10.1186/s12872-022-02990-2.

## Background

Blunt cardiac injury (BCI) includes a wide spectrum of injuries with symptoms ranging from clinically silent arrhythmias to cardiac rupture [1, 2]. Patients with potentially life-threatening chest trauma, particularly those who have sustained high-energy trauma, may present with unclear signs or symptoms; therefore, the identification of patients at increased risk of developing complications due to BCI has been the focus of management [3].

Despite the availability of several diagnostic tests for BCI, none have been established as the definitive standard due to insufficient diagnostic accuracy [1, 3–6]. Among them, electrocardiogram (ECG), troponin I (Tn-I), and troponin T (Tn-T) levels have been used as diagnostic tools for BCI in previous studies [1, 3, 4, 7, 8], however, the utility of other tests for cardiac disease as diagnostic tools for BCI remains unclear.

To develop a diagnostic tool for BCI, additional insight regarding the diagnostic properties of available tests is warranted. Yasue et al. [9] determined that N-terminal pro-B-type natriuretic peptide (NT pro-BNP) is secreted mainly from the left ventricle in healthy adults and patients with left ventricular dysfunction. Additionally, the authors found an increased rate of NT pro-BNP secretion when the left ventricular tension increased. Considering this relationship, Yasue et al. proposed the use of increased serum NT pro-BNP level as a diagnostic and prognostic marker of cardiac dysfunction in patients with congestive heart failure, and ischemic heart disease [10]. NT pro-BNP can be elevated in a variety of clinical setting; Cardiac factors include arrhythmia, myocardial infarction, congestive heart failure, and valvular heart disease, etc. While non-cardiac factors include pulmonary diseases (such as pulmonary arterial hypertension, chronic obstructive pulmonary disease, and pulmonary embolus), sepsis, brain hemorrhage, renal failure, hepatic failure, and old age [11].

Previous studies have investigated the relationship between NT pro-BNP and trauma [4, 6, 10]. Kirchhoff et al. [10] reported the significant correlation of NT pro-BNP levels with clinical signs of multiple organ dysfunction syndrome after multiple injuries at a level 1 trauma center. Additionally, a relationship was observed between NT pro-BNP levels and decreased cardiac index (C.I). The data of this pilot study may indicate the potential value of NT pro-BNP in the diagnosis of post-traumatic cardiac impairment. In 60 patients diagnosed with major trauma, serum NT pro-BNP levels helped predict mortality in these patients [6]. Dogan et al. [4] reported the potential of NT pro-BNP as a marker of BCI in an experimental study of rats with blunt chest trauma, revealing; a significant increase in NT pro-BNP levels in rats with BCI at 5 h after blunt chest trauma. This study suggests the benefits of using NT pro-BNP in the diagnosis of BCI as an adjunct to other tests including Tn-I, ECG, and 2-dimensional (D) echocardiogram.

Here, we hypothesized that indicators for cardiac disease could be helpful in the diagnosis of patients with suspected BCI according to the cardiac injury scale. In addition, we suspected whether C.I, an indicator of cardiac impairment, correlates with traumatic cardiac injury in high-risk intensive care unit (ICU) patients.

Therefore, this study aimed to analyze the use of different indicators for cardiac disease, specifically, NT pro-BNP and C.I, as additional markers in identifying patients with BCI.

## Methods

### Patients

**Fig. 1:**
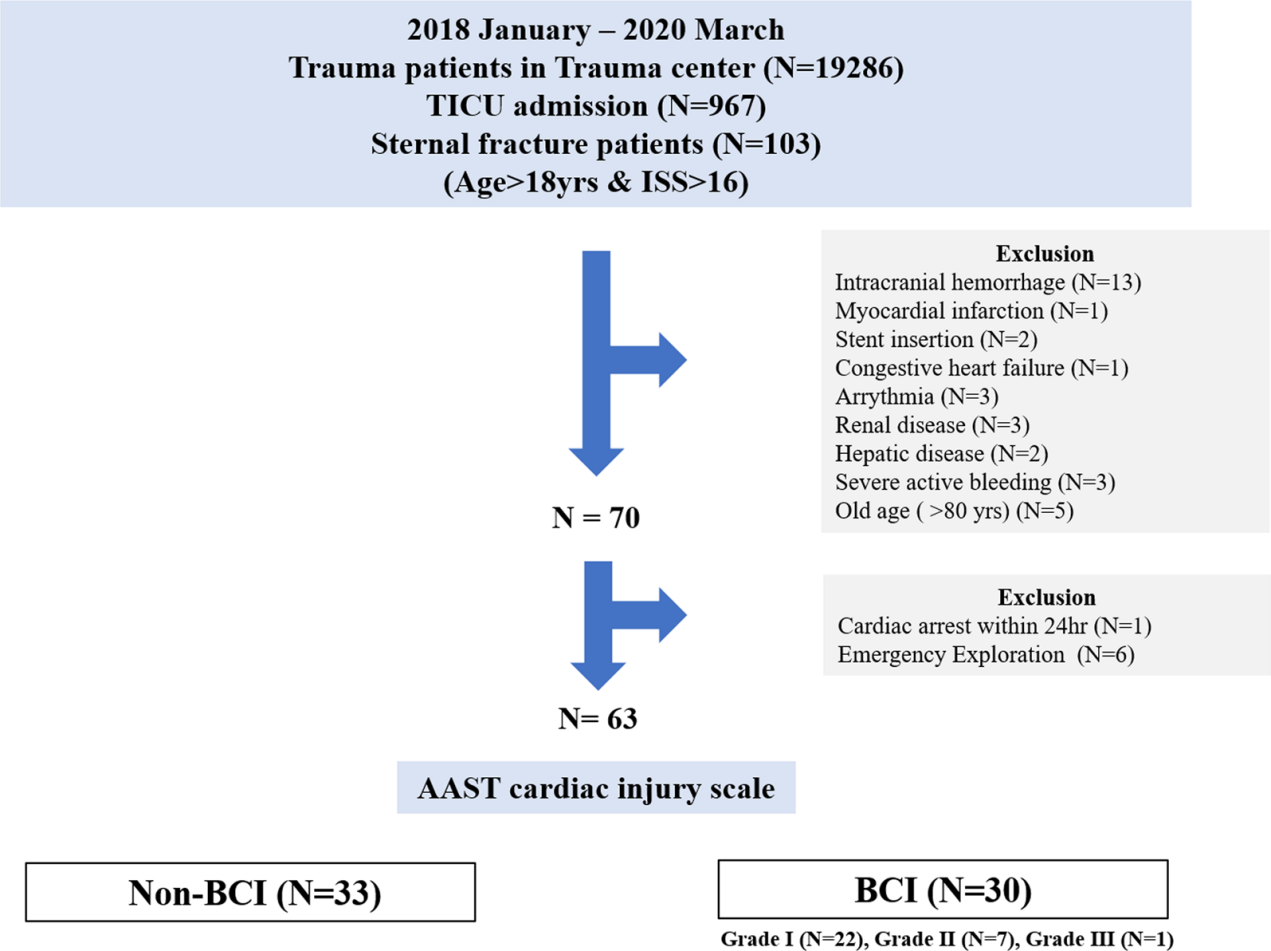
Study design flowchart

In this study, 19,286 patients visited a regional trauma center from January 2018 to March 2020, and 967 patients who were admitted to the traumatic intensive care unit (TICU) had major trauma. Among them, patients with sternum fracture 18 years of age or older, and with an injury severity score (ISS) > 16 who required intensive care were included in this study. According to the American Association for the Surgery of Trauma (AAST) Cardiac Injury Scale [1], patients who met the cardiac injury scale criteria were classified into the BCI group (N = 30), and others were assigned to the non-BCI group (N = 33) The details are shown in Fig. 1.

### Clinical and laboratory assessments

Patients were admitted to the TICU after the administration of initial resuscitation and primary interventions in accordance with standard care. Upon TICU admission, bed rest, pain control, and C.I monitoring were performed. Invasive measurement for the analysis of the pulse contour for continuous C.I monitoring was performed using the FloTrac/EV1000 system (Edwards Lifesciences, Irvine, CA, USA). The FloTrac/EV1000 system is a pulse contour analysis device for continuous cardiac output monitoring. This system is based on continuous arterial blood pressure (femoral artery or radial artery) pulse analysis. The values of C.I was monitored upon TICU admission and performed three times consecutively, and the values were averaged. Intravenous blood sampling was performed for NT pro-BNP, creatinine kinase MB isoenzyme (CK-MB), and Tn-I measurements. Cardiac laboratory assay is based on sandwich immunoassay that use monoclonal antibody use. The Atellica® IM B‑Type natriuretic peptide assay is used as an in vitro diagnostic tool for the quantitative determination of NT pro-BNP in human plasma using the Atellica® IM Analyzer. A normal level of NT pro-BNP is less than 125 pg/mL for 0–80 years of age. The Atellica IM Troponin I assay is a diagnostic tool used in the quantitative measurement of cardiac Tn-I in human serum or plasma samples using the Atellica IM Analyzer. The normal range for Tn-I is between 0 and 0.04 ng/mL. The Atellica™ IM Creatine Kinase MB assay is for in vitro diagnostic use in the quantitative determination of CK‑MB in human serum and plasma using the Atellica™ IM Analyzer. The reference range for the CK-MB level is 0–4.9 ng/mL. Sampling and 12-lead ECG were performed in different time points as follows: immediately after TICU admission and at 24 h and 48 h after trauma.

The institutional review board (IRB) approved this retrospective study and waived the requirement for individual patient consent (IRB approval number: 2020-04-063).

### Statistical analysis

Categorical variables are reported as absolute numbers or percentages, which were analyzed using the Fisher’s exact or Chi-square test. Continuous variables are presented as mean ± standard deviation (SD) or as median and interquartile range (IQR). Parametric and non-parametric data were analyzed using Student’s t-test and Wilcoxon test, respectively. Statistical significance was set at *p* < 0.05. The data were analyzed using SAS/STAT software (version 9.4; SAS Institute Inc., Cary, NC, USA). The efficacies of NT pro-BNP, C.I and lactate levels in predicting BCI were analyzed using areas under the receiver operating characteristic (ROC) curves.

## Results

Among 103 trauma patients, 40 patients were excluded from the study: 33 patients due to factors that may affect NT pro-BNP, including intracranial hemorrhage (N = 13), previous myocardial infarction (N = 1), previous coronary artery stent insertion (N = 2), congestive heart failure (N = 1), arrhythmia (N = 3), renal disease (N = 3), hepatic disease (N = 2), severe active bleeding (N = 3), and old age (> 80 years) (N = 5). Six patients were excluded because of cardiac exploration due to traumatic hemopericardium, which required clear surgical treatment or procedures at the time of admission, These patients had definite cardiac structure injury that required cardiac operation, which might affect diagnostic test results (cardiac incision, cardiopulmonary bypass use, postoperative acute kidney injury etc.). One patient was excluded due to cardiac arrest within 24 h. Finally, 63 patients were included in the study.

The baseline characteristics of the patients are shown in Table 1. The median ages of the patients were 58 (52–69), and 60 (45–69) years in the non-BCI and BCI groups (*p* = 0.77), respectively. Both groups showed male predominance (24 (72.7%) in the non-BCI group vs. 24 (80.0%) in the BCI group, *p* = 0.49); No significant difference was observed in the mean initial systolic blood pressure and Glasgow coma scale score between the two groups (121 ± 34.2 vs. 112 ± 29.3 mmHg, *p* = 0.20 and 14 ± 0.3 vs. 13 ± 2.3, *p* = 0.77, respectively). Preoperative shock status was higher in the BCI group; however, there was no statistical significance (3 (9.1%) in the non-BCI group vs. 8 (26.7%) in the BCI group, *p* = 0.06) In addition, there was no difference in the initial blood loss between the two groups (*p* = 0.56). The number of red blood cell transfusions also showed no statistical difference between the groups (1.6 ± 1.7 in the non-BCI group vs. 1.9 ± 1.8 in the BCI group, *p* = 0.50) Furthermore, no significant statistical difference was observed in the mean chest Abbreviated Injury Scale (2.8 ± 0.65 vs*.* 3.0 ± 0.8, *p* = 0.08) and the mean ISS between the non-BCI and BCI groups (19.3 ± 2.6 vs. 22.8 ± 5.9, *p* = 0.06).

**Table 1:** Baseline characteristics of patients

Characteristics	Non-BCI (N = 33)	BCI (N = 30)	*p* value
Age (years, median, IQR)	58 (52–69)	60 (45–69)	0.77
Gender (male, n, %)	24 (72.7)	24 (80.0)	0.49
Height (cm, mean ± SD)	169.5 ± 7.6	168.3 ± 8.7	0.57
Weight (kg, mean ± SD)	67.1 ± 10.1	67.6 ± 9.7	0.84
Initial SBP (mmHg, mean ± SD)	121 ± 34.2	112 ± 29.3	0.20
GCS score (mean ± SD)	14 ± 0.3	13 ± 2.3	0.77
Intubation (n, %)	3 (9.1)	7 (23.3)	0.12
Preoperative shock status (n, %)	3 (9.1)	8 (26.7)	0.06
Initial blood loss			0.56
Class I (< 750 ml, n, %)	25 (75.8)	21 (70.0)	
Class II (750–1500 ml, n, %)	7 (21.2)	9 (30.0)	
Class III (1500–2000 ml, n, %)	1 (3.0)	0 (0.0)	
No. red blood cell transfusion (mean ± SD)	1.6 ± 1.7	1.9 ± 1.8	0.50
Hypertension (n, %)	10 (30.3)	13 (43.3)	0.28
Diabetes (n, %)	3 (9.1)	3 (10.0)	0.90
Current smoking (n, %)	12 (36.4)	12 (40.0)	0.76
COPD (n, %)	2 (6.1)	3 (10.0)	0.56
Stroke (n, %)	1 (3.0)	3 (10.0)	0.25
Initial hemoglobin (g/dl, mean ± SD)	12.8 ± 1.8	11.9 ± 1.9	0.07
Initial eGFR (mL/min/BSA, mean ± SD))	97.6 ± 18.8	96.2 ± 18.5	0.75
Previous operation (n, %)	2 (6.1)	3 (10.0)	0.60
Transfer from another hospital (n, %)	14 (42.4)	12 (40.0)	0.84
Chest AIS (mean ± SD)	2.8 ± 0.7	3.0 ± 0.8	0.08
ISS (mean ± SD)	19.3 ± 2.6	22.8 ± 5.9	0.06

BCI, Blunt cardiac injury; IQR, Interquartile range; SD, Standard deviation; SBP, Systemic blood pressure; GCS, Glasgow Coma Scale; No, Number; COPD, Chronic obstructive pulmonary disease; eGFR, Estimated glomerular filtration rate; AIS, Abbreviated injury scale; ISS, Injury severity score

The causes of injury and combined injury are detailed in Table 2. Automobile-related injuries were the most common causes of injury in the non-BCI group (39.4%). Automobile-related injuries (30.0%) and falls (30.0%) were the most common cause of injury in the BCI group. However, no significant difference was observed regarding the cause of injury between the two groups (*p* = 0.09). Injury by animal damage was present in one case in each groups (3.0% in the non-BCI group vs. 3.3% in the BCI group); both patients were hit by a cow horn. When comparing associated injuries, multiple rib fractures were found to be the most common in both groups; however, no significant differences was observed (87.9% vs. 93.3%, *p* = 0.46). In addition, there was no statistically significant statistical difference in flail chest between the groups (12.1% in the non-BCI group vs. 10.0% in the BCI group, *p* = 0.78).

**Table 2:** Cause of injury and combined injury

Characteristics	Non-BCI (N = 33)	BCI (N = 30)	*p* value
Cause of injury			0.09
Auto-pedestrian (n, %)	0 (0.0)	1 (3.3)	
Automobile (n, %)	13 (39.4)	9 (30.0)	
Motorcycle (n, %)	1(3.0)	7 (23.3)	
Fall down (n, %)	8 (24.2)	9 (30.0)	
Other vehicles^a^ (n, %)	4 (12.1)	2 (6.7)	
Animal (cow) damage (n, %)	1(3.0)	1 (3.3)	
Entrapment of machine (n, %)	6 (18.2)	1 (3.3)	
Associated injuries			
Multiple rib fracture (n, %)	29 (87.9)	29 (93.3)	0.46
Flail chest (n, %)	4 (12.1)	3 (10.0)	0.78
Lung contusion (n, %)	10 (30.3)	8 (26.7)	0.75
Hemo-pneumothorax (n, %)	7 (21.2)	11 (36.7)	0.17
Aortic injury (n, %)	1(3.0)	3 (10.0)	0.25

*BCI* Blunt cardiac injury

^a^Bike, stretch car or cultivator

The diagnostic test details are shown in Table 3. Regarding the relationship between sternum fracture severity and BCI, no significant relationship was observed between BCI with displaced sternal fracture and severity (48.5% vs. 46.7%, *p* = 0.88 and 6.1% vs. 13.3%, *p* = 0.32, respectively). Additionally, there was no statistically significant difference in the presence of retrosternal hematoma between the two groups (45.5% in the non-BCI group vs. 53.3% in the BCI group, *p* = 0.53).

**Table 3:** Diagnostic test details

Characteristics	Non-BCI (N = 33)	BCI (N = 30)	*p* value
Displaced sternal fracture (n, %)	16 (48.5)	14 (46.7)	0.88
Severe displaced sternal fracture^a^ (n, %)	2 (6.1)	4 (13.3)	0.32
Retrosternal hematoma (n, %)	15 (45.5)	16 (53.3)	0.53
Electrocardiogram			
Normal sinus rhythm (n, %)	33 (100.0)		
Sinus tachycardia (n, %)		13 (43.3)	
T wave abnormality (n, %)		4 (13.3)	
T inversion (n, %)		1 (3.3)	
Right bundle branch block (n, %)		2 (6.7)	
Left anterior fascicular block (n, %)		2 (6.7)	
Borderline ST elevation (n, %)		1 (3.3)	
Atrioventricular block (n, %)		1 (3.3)	
Premature ventricular contraction (n, %)		4 (13.3)	
Premature atrial contraction (n, %)		2 (6.7)	

*BCI* Blunt cardiac injury

^a^Displacement > 5 mm

Regarding the initial ECG results of BCI, ECG change and non-specific changes, including sinus tachycardia, were present in 43.3% of the patients. T wave abnormality (13.3%) and premature ventricular contraction (13.3%) were followed by sinus tachycardia, and borderline ST elevation was observed in 1 patient (3.3%). Atrial fibrillation (AF) on ECG occurred in 2 patients on hospital day (HD) 2. One patient with sinus tachycardia on ECG changed to AF on HD 2 and one patient with frequent premature ventricular contraction patient also changed to AF on HD 2. Additionally, atrioventricular block occurred in one patient (3.3%) from BCI.

**Fig. 2:**
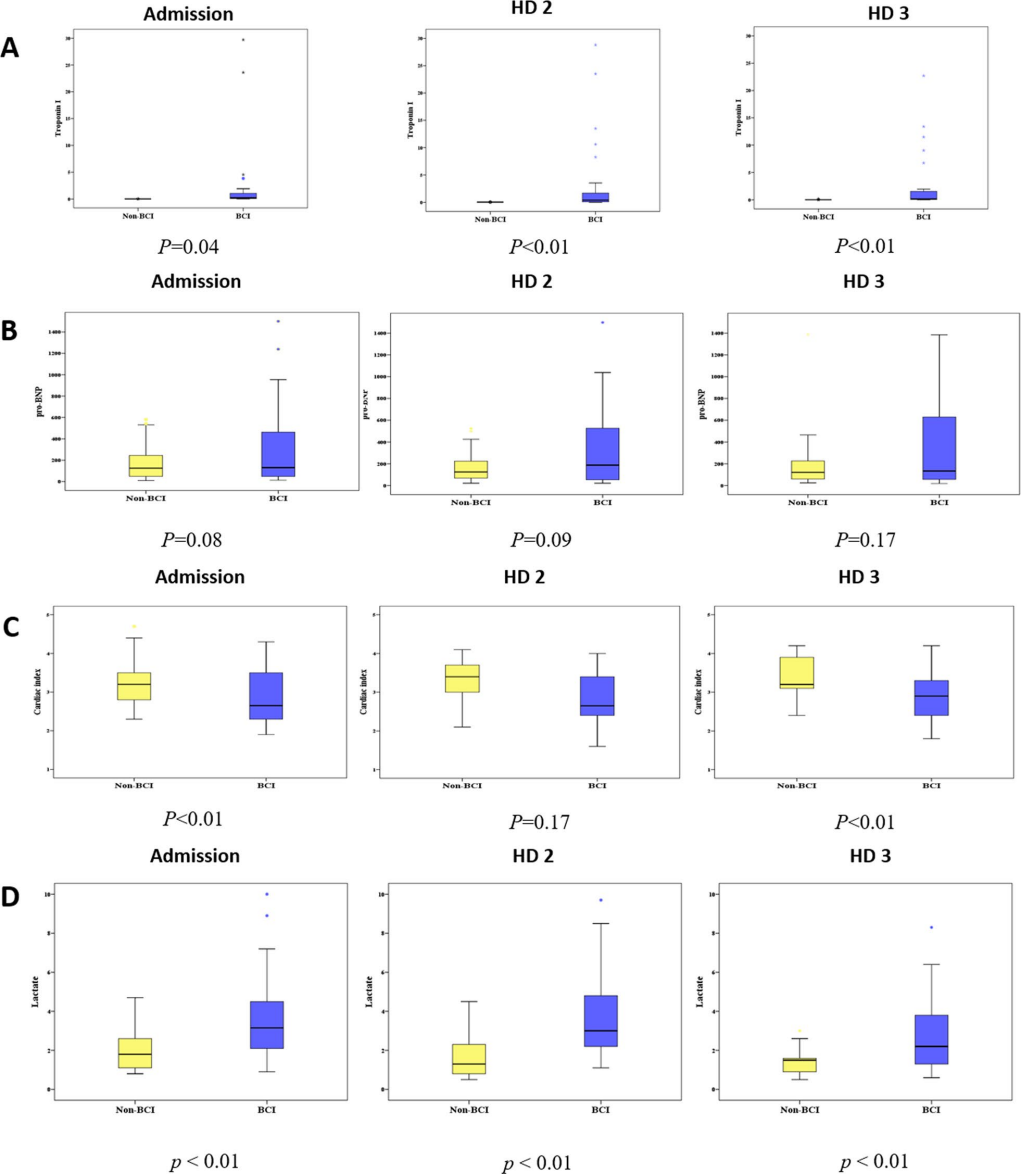
**A** Serial change of serum Troponin-I. **B** Serial change of serum NT pro-BNP. **C** Serial change of cardiac index. **D** Serial change of serum lactate

Between the two groups, the median Tn- I levels were showed that it was statistically significantly lower in the non-BCI group than in the BCI group (0.0 (0.0–0.0) vs. 0.2 (0.0–1.0) pg/mL, *p* = 0.04, 0.0 (0.0–0.0) vs*.* 0.4 (0.0–1.6) pg/mL*, p* < 0.01, and 0.0 (0.0–0.0) vs. 0.2 (0.0–1.4) pg/mL, *p* < 0.01, respectively) (Fig. 2A). No significant differences were observed in the median CK-MB levels between the non-BCI and BCI groups on admission and HD 2 (8.5 (4.45–11.3) vs. 8.7 (5.5–17.3) ng/mL, *p* = 0.58, and, 5.8 (2.3–18.1) vs. 12.2 (4.4–22.9) ng/mL, *p* = 0.16, respectively); however, statistically higher CK-MB values were observed in the BCI group on HD 3 (3.2 (1.8–10.2) vs.6.9 (2.1–16.4) ng/mL, *p* < 0.01). The detailed values are shown in Sup. 1.

No significant difference was observed in the median NT pro-BNP values on admission, HD 2, and HD 3 between the non-BCI and BCI groups (125 (49–245) vs. 130 (47–428) pg/mL, *p* = 0.08; 124 (68–224) vs*.* 187 (55–519) pg/mL*, p* = 0.09; and 121 (59–225) vs. 133 (56–600) pg/mL, *p* = 0.17, respectively) (Fig. 2B). On the contrary, significantly higher in the median C.I was observed on admission and HD 3 in the non-BCI (3.2 (2.8–3.5) vs*.* 2.6 (2.3–3.5) L/min/m^2^, *p* < 0.01 and 3.2 (3.1–3.9) vs*.* 2.9 (2.4–3.2) L/min/m^2^, *p* < 0.01, respectively); however, no significant difference was observed on HD 2 (3.4 (3.0–3.7) vs*.* 2.6 (2.4–3.4) L/min/m^2^, *p* = 0.17) (Fig. 2C). Furthermore, the median lactate levels upon admission HD 2, and HD 3 were significantly different between the non-BCI and BCI groups (1.8 (1.1–2.6) vs*.* 3.1 (2.1–4.4) mmol/L, *p* < 0.01, 1.3 (0.8–2.3) vs*.* 3.0 (2.2–4.7) mmol/L, *p* < 0.01, and 1.5 (0.9–1.5) vs*.* 2.2 (1.3–3.7) mmol/L, *p* < 0.01, respectively) (Fig. 2D).

**Fig. 3:**
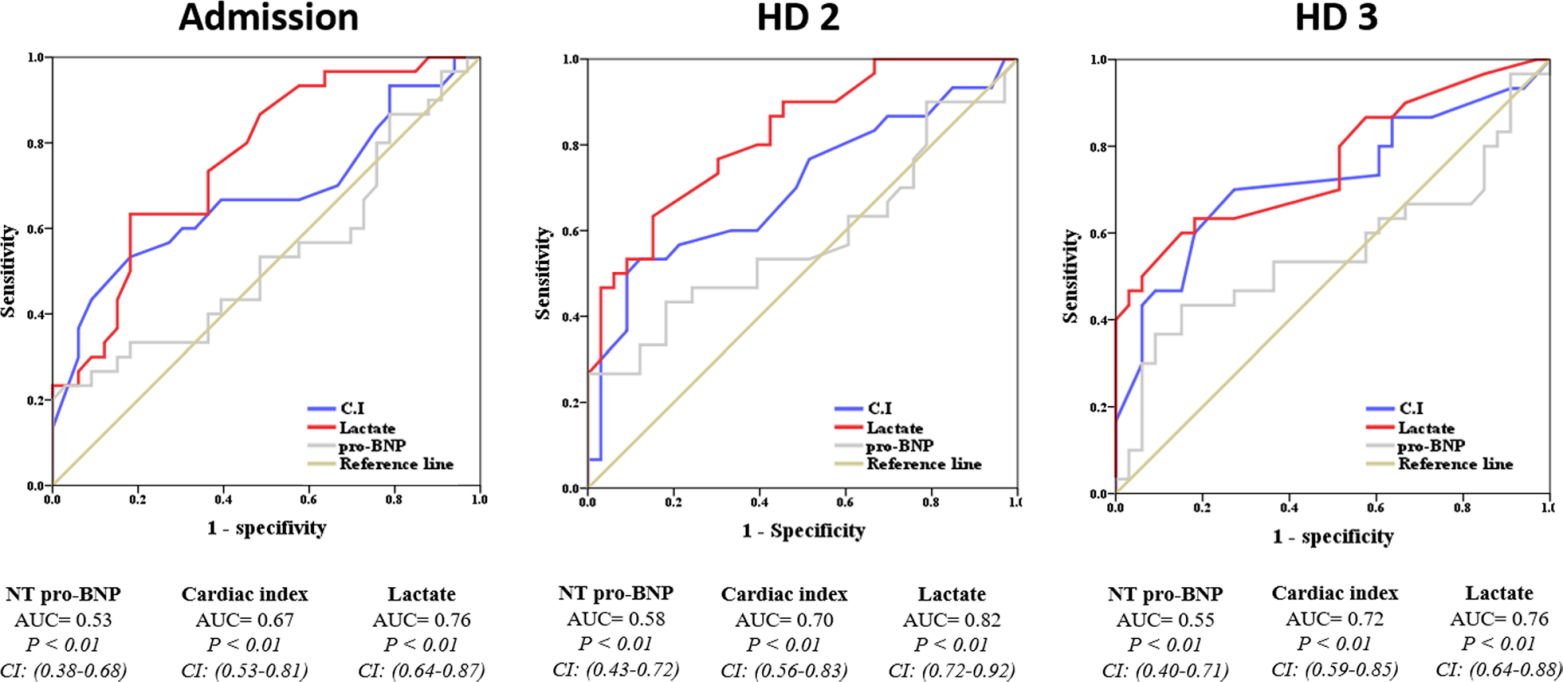
ROC curve for diagnostic test

The areas under the ROC curve of C.I on admission, HD2, and HD3 were 0.67, 0.70 and 0.72, respectively. In addition, the areas under the ROC curve of lactate on admission, HD2, and HD3 were 0.76, 0.82 and 0.76, respectively (Fig. 3). The point containing the maximum Youden index in the ROC curve was defined as the ideal cutoff point. The ideal cutoff point at each time point is shown in Sup. 2.

Table 4 shows the 2D echocardiography results in the BCI group. All 30 patients underwent 2D echocardiography, and mean ejection fraction was 56.8 ± 2.8%. Two patients had regional wall motion abnormality; one patient had wall motion abnormality in the lateral inferior wall, and one patient had it in the apex. There was one patient with mild to moderate tricuspid regurgitation and one patient with severe tricuspid regurgitation. A patient with severe tricuspid regurgitation was diagnosed by 2D echocardiography on HD 3, and tricuspid valve replacement was performed on HD 4. One patient with severe mitral regurgitation with wall motion abnormality of the lateral inferior wall, underwent mitral valve replacement on HD 4. Both patients who underwent cardiac surgery were discharged without complications.

**Table 4:** 2-Dimensional echocardiography results

Characteristics	BCI (N = 30)
Echocardiography (n, %)	30 (100)
Ejection fraction (%, mean ± SD)	56.8 ± 2.8
Wall abnormality (n, %)	2 (6.6, lateral, inferior wall [1], apex [1])
Valve involvement (n, %)	14 (46.7)
Mild tricuspid regurgitation (n, %)	8 (26.7)
Mild to moderate tricuspid regurgitation (n, %)	1 (3.3)
Severe tricuspid regurgitation (n, %)	1 (3.3)
Mild mitral regurgitation (n, %)	3 (10.0)
Severe mitral regurgitation	1 (3.3)
Mean right ventricular systolic pressure (mmHg, mean ± SD)	33.3 ± 14.8

*BCI* Blunt cardiac injury, *SD* Standard deviation

The postoperative outcomes are shown in Table 5. Skeletal part surgery was performed the most for the accompanying injury, and there was no difference regarding the operation of the accompanying injury between non-BCI and BCI groups (*p* = 0.92). A significantly longer time was observed in the total ICU stay and total ventilator care time in the BCI group (*p* < 0.01for both); however, the length of stay was comparable between the two groups (20.7 ± 16.8 in the non-BCI group vs. 21.8 ± 21.3 days in the BCI group, *p* = 0.83). In-hospital mortality occurred in two patients in the BCI group, and both died from ventilator-related pneumonia.

**Table 5:** Postoperative outcomes

Characteristics	Non-BCI (N = 33)	BCI (N = 30)	*p* value
Surgical treatment (n, %)	15 (45.5)	14 (46.7)	0.92
Facial part (n, %)	2 (13.3)	1 (7.1)	
Chest part (n, %)	1 (6.7)	2 (14.3)	
Spine part (n, %)	3 (20.0)	5 (35.7)	
Skeletal part (n, %)	9 (60.0)	6 (42.9)	
Length of stay (day, mean ± SD)	20.7 ± 16.8	21.8 ± 21.3	0.83
Total ICU stay (day, mean ± SD)	5.48 ± 86.7	10.29 ± 89.5	< 0.01
Total ventilator care (min, mean ± SD)	23.3 ± 65.2	149.8 ± 189.3	< 0.01
Reintubation (n, %)	0 (0.0)	6 (20.0)	< 0.01
Transferred (n, %)	25 (75.8)	17 (56.7)	0.10
In-hospital mortality (n, %)	0 (0.0)	2 (6.7)	0.13

*BCI* Blunt cardiac injury, *IQR* Interquartile range, *SD* Standard deviation, *ICU* Intensive care unit

## Discussion

BCI includes a wide spectrum of clinical symptoms. The decelerating force leads to mechanical cardiac injuries, including valvular, septal, ventricular, atrial, or septal wall damage. Additionally, relatively mild myocardial contusion may lead to other adverse cardiac events, such as mild arrhythmias [1–3]. Considering the numerosity of complications, careful monitoring is warranted in patients with suspected BCI. However, diagnosis of BCI remains a challenge due to the absence of a clear definition and a gold standard diagnostic test [1–3, 12–14]. Managing physicians should be aware that chest injuries including pulmonary contusion, sternum fracture, and multiple rib fractures are possible indicators of BCI. Therefore, the role of complete diagnosis after typical trauma mechanisms should not be underestimated even in patients whose clinical condition is not indicative of severe injury [1].

BCI arises from a variety of injuries, often due to high-speed impact. The most common causes of injury is motor vehicle accidents; in addition, bicycle accidents, falls, sports-related injuries, and even assaults can also result in the BCI [2, 3, 15, 16].

A few clinical signs or symptoms are specific of BCI. The most common clinical sign associated with BCI is chest pain, which may or may not be anginal in nature, and it is usually a result of associated chest trauma [17]. 2D echocardiography and ECG may indicate damage to the tissue architecture and subsequent complications, however, information on specific cellular damage within the heart muscle requires the measurement of cardiac muscle-specific proteins such as Tn-T, Tn-I, or creatine kinase muscle [1, 6, 18]. Tissue damage always occurs with cellular damage, but cellular damage does not require tissue damage; therefore, a combination of tests is usually used [1]. However, ECG, cardiac enzymes, and echocardiography in the diagnosis of BCI remains controversial [3]. Level I evidence supports that all patients with suspicious BCI should obtain 12-lead ECG upon admission or at the emergency room [8]. The use of ECG changes has demonstrated a sensitivity, specificity, and negative predictive value of 100%, 47%, and 90% respectively, in the detection of BCI-related complications that require treatment; these changes include sinus tachycardia, bradycardia, conduction delays, or atrial or ventricular dysrhythmias [12]. Additionally, Tn-I and Tn-T have been recently used to screen for BCIs. In 26 patients diagnosed with BCI using ECG or echocardiographic criteria, the sensitivity of Tn-I and Tn-T was 23% and 12%, respectively, while the specificity was 97% and 100%, respectively [19]. A report by Salim et al. [7] found that patients with normal ECG and serial values of Tn-I had no significant BCI-related complications. Furthermore, they found that abnormal ECG and elevated Tn-I had 100% sensitivity and 62% positive predictive value in diagnosing clinically significant BCI. Based on the results ofT these studies, the clinical value of Tn-I, when simultaneously performed with ECG, has increased. Although, there are still no definite guidelines, the ECG-based AAST Cardiac Injury Scale is widely used.

Traumatic sternal fractures indicate a high risk of the presence of BCI [14]. Based this risk, our study was limited to patients with sternal fractures from among a wide range of patients admitted to the TICU. Absolute bed rest was the principle for accurate monitoring. We investigated the possible adjuncts to other diagnostic tests of BCI through serial monitoring and candidate biomarker measurements.

In this study, no significant difference was observed in the length of stay between the two groups; however, significant differences were observed in total TICU stay and total ventilator care. No significant difference was observed between the ISS of the two groups; however, the BCI group may require more cardiac monitoring and long-term intensive care compared with the non-BCI group. In addition, 2D-echocardiography data did not show poor cardiac output, ie, poor EF in the BCI group, and no patient underwent additional procedures except for two patients. These results indicate a favorable prognosis for patients diagnosed with BCI group without cardiac structural injury.

No relationship was observed between NT pro-BNP and BCI which was inconsistent with the results of previous studies. The study of Dogan et al. [4] is experimental study that showed the relationship between NT-Pro BNP and BCI using rats. Unlike our study, there was no clear standard for BCI which may be the reason for the different. conclusions reached Kirchhoff et al. performed a prospective cohort study, showing a relationship between NT-Pro BNP and multiple organ dysfunction syndrome. This pilot study inferred the association between NT pro-BNP levels and cardiac impairment by correlating that increased NT pro-BNP levels and decreased C.I were tolerant. In this study, the authors concluded that NT pro-BNP levels and decreased C.I were related. In our study, a significant decrease in C.I was observed in the BCI group on admission and HD 3. However, no significant difference in C.I value was observed on HD 2. Therefore, since this study does not show concordance in consecutive results; it cannot be concluded that there is a relationship between value of C.I and BCI. Here, the difference is that the decrease in the C.I was studied not for cardiac impairment, but for the association of BCI with specific diagnostic criteria. What is inconsistent with previous studies and the results of this study are that there is no clear criteria for BCI in trauma patients, suggesting that more research is needed on this issue.

Additionally, the lactate level was included in the analyses by examining the correlation of lactate level indicators with BCI. In this study, the most important indices of severity in trauma were the ISS score, preoperative shock status, number of red blood cell transfusion, and initial blood loss, none of which showed no statistical difference between the two groups. These variables are related to lactate levels. Therefore, we conclude that BCI may be associated with elevated lactate levels unless these variables act as confounding factors. Since a few studies have been reported on this topic, future researches should focus on the relationship between BCI and lactate levels.

This study has some limitations. First, it was a single-center retrospective study that included a small number of patients. Second, since no accurate diagnostic criteria for BCI exists, we used the AAST cardiac injury scale as the diagnostic criterion of BCI, except for patients with emergency surgical procedures. Therefore, this study seems to be meaningful only for the association between mild traumatic cardiac injury focused on ECG changes and the values presented in this study. Third, the postoperative results are highly influenced by other accompanying injuries as the criteria were based on major trauma patients who were admitted to the TICU with an ISS score of 16 or higher. Finally, this study investigated diagnostic adjuvant tools; therefore, it does not influence the treatment of patients and has no influence on patient survival and treatment direction.

## Conclusions

In this study, we investigated the competence of NT pro-BNP and C.I as adjunctive diagnostic tools for BCI. Consecutive values of NT pro-BNP and C.I showed no correlation with ECG-based BCI diagnosis. However, lactate level may help in the early recognition of BCI as an adjunctive tool. It is worth underlining that was a hypothesis-generating study for BCI diagnosis; therefore, more research is needed to evaluate the accurate relevance of the study results.

## Supplementary Information

**Additional file 1. Table S1.** Diagnostic test details.

**Additional file 2. Table S2.** Details of diagnostic values.

## Data Availability

All data generated or analyzed during this study are included in this published article and its Additional file 1: Table S1 and Additional file 2: Table S2.

## References

[1] Van Lieshout EMM, Verhofstad MHJ, Van Silfhout DJT, Dubois EA (2020). Diagnostic approach for myocardial contusion: a retrospective evaluation of patient data and review of the literature. Eur J Trauma Emerg Surg.

[2] Mattox KL, Flint LM, Carrico CJ, Grover F, Meredith J, Morris J, Rice C, Richardson D, Rodriquez A, Trunkey DD (1992). Blunt cardiac injury. J Trauma.

[3] Schultz JM, Trunkey DD (2004). Blunt cardiac injury. Crit Care Clin.

[4] Dogan H, Sarikaya S, Neijmann ST, Uysal E, Yucel N, Ozucelik DN, Okuturlar Y, Solak S, Sever N, Ayan C (2015). N-terminal pro-B-type natriuretic peptide as a marker of blunt cardiac contusion in trauma. Int J Clin Exp Pathol.

[5] Marcolini EG, Keegan J (2015). Blunt cardiac injury. Emerg Med Clin N Am.

[6] Qian A, Zhang M, Zhao G (2015). Dynamic detection of N-terminal pro-B-type natriuretic peptide helps to predict the outcome of patients with major trauma. Eur J Trauma Emerg Surg.

[7] Salim A, Velmahos GC, Jindal A, Chan L, Vassiliu P, Belzberg H, Asensio J, Demetriades D (2001). Clinically significant blunt cardiac trauma: role of serum troponin levels combined with electrocardiographic findings. J Trauma.

[8] Pasquale M, Fabian TC (1998). Practice management guidelines for trauma from the Eastern Association for the Surgery of Trauma. J Trauma.

[9] Yasue H, Yoshimura M, Sumida H, Kikuta K, Kugiyama K, Jougasaki M, Ogawa H, Okumura K, Mukoyama M, Nakao K (1994). Localization and mechanism of secretion of B-type natriuretic peptide in comparison with those of A-type natriuretic peptide in normal subjects and patients with heart failure. Circulation.

[10] Kirchhoff C, Leidel BA, Kirchhoff S, Braunstein V, Bogner V, Kreimeier U, Mutschler W, Biberthaler P (2008). Analysis of N-terminal pro-B-type natriuretic peptide and cardiac index in multiple injured patients: a prospective cohort study. Crit Care.

[11] Burke MA, Cotts WG (2007). Interpretation of B-type natriuretic peptide in cardiac disease and other comorbid conditions. Heart Fail Rev.

[12] Healey MA, Brown R, Fleiszer D (1990). Blunt cardiac injury: Is this diagnosis necessary?. J Trauma.

[13] Brathwaite CE, Rodriguez A, Turney SZ, Dunham CM, Cowley R (1990). Blunt traumatic cardiac rupture. A 5-year experience. Ann Surg.

[14] Hanschen M, Kanz KG, Kirchhoff C, Khalil PN, Wierer M, van Griensven M, Laugwitz KL, Biberthaler P, Lefering R, Huber-Wagner S (2015). Blunt cardiac injury in the severely injured—a retrospective multicentre study. PLoS ONE.

[15] Bu'Lock FA, Prothero A, Shaw C, Parry A, Dodds CA, Keenan J, Forfar JC (1994). Cardiac involvement in seatbelt-related and direct sternal trauma: a prospective study and management implications. Eur Heart J.

[16] Crown LA, Hawkins W (1997). Commotio cordis: clinical implications of blunt cardiac trauma. Am Fam Physician.

[17] Snow N, Richardson JD, Flint LM (1982). Myocardial contusion: implications for patients with multiple traumatic injuries. Surgery.

[18] Greenberg MD, Rosen CL (1999). Evaluation of the patient with blunt chest trauma: an evidence based approach. Emerg Med Clin N Am.

[19] Bertinchant JP, Polge A, Mohty D, Nguyen-Ngoc-Lam R, Estorc J, Cohendy R, Joubert P, Poupard P, Fabbro-Peray P, Monpeyroux F (2000). Evaluation of incidence, clinical significance, and prognostic value of circulating cardiac troponin I and T elevation in hemodynamically stable patients with suspected myocardial contusion after blunt chest trauma. J Trauma.

