# Supplementary material for: Serum pro-B-type natriuretic peptide levels and cardiac index as adjunctive tools of blunt cardiac injury
Source: BMC Cardiovasc Disord. 2023 Feb 10;23:81. doi: 10.1186/s12872-022-02990-2 (PMC9912581; doi:10.1186/s12872-022-02990-2)
Supplement: Supplementary file 1 — Additional file 1. Table S1. Diagnostic test details. [file 12872_2022_2990_MOESM1_ESM.docx]

**Supplement Table 1. Diagnostic test details**

| **Characteristics** | **Non-BCI (N = 33)** | **BCI (N = 30)** | ***p*-value** |
| --- | --- | --- | --- |
| Tn-I (Admission, pg/mL, median, IQR) | 0.0 (0.0 – 0.0) | 0.2 (0.0 – 1.0) | 0.04 |
| Tn-I (HD 2, pg/mL, median, IQR) | 0.0 (0.0 – 0.0) | 0.4 (0.0– 1.6) | <0.01 |
| Tn-I (HD 3, pg/mL, median, IQR) | 0.0 (0.0 – 0.0) | 0.2 (0.0– 1.4) | <0.01 |
| NT pro-BNP (Admission, pg/mL median, IQR) | 125 (49–245) | 130 (47 – 428) | 0.08 |
| NT pro-BNP (HD 2, pg/mL, median, IQR) | 124 (68– 224) | 187 (55 – 519) | 0.09 |
| NT pro-BNP (HD 3, pg/mL, median, IQR) | 121 (59– 225) | 133 (56 – 600) | 0.17 |
| C.I (Admission, L/min/m^2^ , median, IQR) | 3.2 (2.8 – 3.5) | 2.6 (2.3 – 3.5) | <0.01 |
| C.I (HD 2, L/min/m^2^ , median, IQR) | 3.4 (3.0 – 3.7) | 2.6 (2.4 – 3.4) | 0.17 |
| C.I (HD 3, L/min/m^2^ , median, IQR) | 3.2 (3.1 – 3.9) | 2.9 (2.4 – 3.2) | <0.01 |
| CK-MB(Admission, ng/mL, median, IQR) | 8.5 (4.45 – 11.3) | 8.7 (5.5 – 17.3) | 0.58 |
| CK-MB (HD 2, ng/mL, median, IQR) | 5.8 (2.3– 18.1) | 12.2 (4.4 – 22.9) | 0.16 |
| CK-MB (HD 3, ng/mL, median, IQR) | 3.2 (1.8 – 10.2) | 6.9 (2.1 – 16.4) | <0.01 |
| Lactate (Admission, mmol/L, median, IQR) | 1.8 (1.1 – 2.6) | 3.1 (2.1 – 4.4) | <0.01 |
| Lactate (HD 2, mmol/L, median, IQR) | 1.3 (0.8 – 2.3) | 3.0 (2.2 – 4.7) | <0.01 |
| Lactate (HD 3, mmol/L, median, IQR) | 1.5 (0.9 – 1.5) | 2.2 (1.3– 3.7) | <0.01 |
| BCI, blunt cardiac injury; IQR, interquartile range; Tn-I, Troponin I; HD, hospital day; NT pro-BNP, N-terminal pro-B-type natriuretic peptide; C.I, cardiac index; CK-MB, creatine kinase MB isoenzyme | | | |
