# Supplementary material for: Serum pro-B-type natriuretic peptide levels and cardiac index as adjunctive tools of blunt cardiac injury
Source: BMC Cardiovasc Disord. 2023 Feb 10;23:81. doi: 10.1186/s12872-022-02990-2 (PMC9912581; doi:10.1186/s12872-022-02990-2)
Supplement: Supplementary file 2 — Additional file 2. Table S2. Details of diagnostic values. [file 12872_2022_2990_MOESM2_ESM.docx]

**Supplement Table 2. Details of diagnostic values**

|  | **AUC (95 % CI)** | ***p-value*** | **Cut-off** | **Sensitivity** | **Specificity** | **PPV** | **NPV** | |
| --- | --- | --- | --- | --- | --- | --- | --- | --- |
| NT pro-BNP (Admission) | 0.53(0.38-0.68) | *< 0.01* | 580 | 23.3% | 97.0% | 87.5% | 58.2% | |
| NT pro-BNP (HD2) | 0.58(0.43-0.72) | *< 0.01* | 526.7 | 26.7% | 100.0% | 100.0% | 60.0% | |
| NT pro-BNP (HD3) | 0.55(0.40-0.71) | *< 0.01* | 278.4 | 43.3% | 84.8% | 72.2% | 62.2% | |
| Cardiac index (Admission) | 0.67 (0.53-0.81) | *< 0.01* | 2.7 | 53.3% | 81.8% | 72.7% | 65.9% | |
| Cardiac index (HD2) | 0.70 (0.56-0.83) | *< 0.01* | 2.7 | 53.3% | 87.9% | 80.0% | 67.4% | |
| Cardiac index (HD3) | 0.72 (0.59-0.85) | *< 0.01* | 3.1 | 70.0% | 72.7% | 70.0% | 72.7% | |
| Lactate (Admission) | 0.76 (0.64-0.87) | *< 0.01* | 2.9 | 63.3% | 81.8% | 76.0% | 71.1% | |
| Lactate (HD2) | 0.82 (0.72-0.92) | *< 0.01* | 2.4 | 63.3% | 84.8% | 79.2% | 71.8% | |
| Lactate (HD3) | 0.76 (0.64-0.88) | *< 0.01* | 1.9 | 63.3% | 81.8% | 76.0% | 71.1% | |
| AUC, area under curve; C.I. confidence interval; HD, hospital day; PPV, positive predictive value; NPV, negative predictive value; NT pro-BNP, N-terminal pro-B-type natriuretic peptide | | | | | | | |  |
